# Epigallocatechin Gallate From Tea Delays Oocyte Aging After Ovulation in Mice

**DOI:** 10.1002/fsn3.72324

**Published:** 2026-09-05

**Authors:** Jing Lin, Hua‐Chao Gao, Li‐Jun Li, Miao Hu, Li‐Fan Guo, Hong‐Yan Guo, Xiao‐Wen Jin, Peng Xu, Zhao‐Jia Ge, Lei Zhao

**Affiliations:** ^1^ College of Horticulture Qingdao Agricultural University Qingdao People's Republic of China; ^2^ College of Life Science Qingdao Agricultural University Qingdao People's Republic of China; ^3^ College of Animal Science and Technology Qingdao Agricultural University Qingdao People's Republic of China; ^4^ College of Life Science Sichuan Agricultural University Ya'an People's Republic of China; ^5^ Lvliang First People's Hospital, Lvliang Hospital of Shanxi Medical University Lvliang China; ^6^ Key Laboratory of Chemical Biology and Molecular Engineering of Ministry of Education, School of Life Science Shanxi University Taiyuan China

**Keywords:** EGCG, oocyte, postovulatory aging, tea

## Abstract

In clinic, once the ovulated oocytes cannot be fertilized timely, they begin to undergo post‐ovulatory aging, which impacts the embryo development and offspring health. Until now, there are no better methods to delay post‐ovulatory oocyte aging. Epigallocatechin gallate (EGCG) is the most abundant bioactive component of tea polyphenols that are beneficial for alleviating the deleterious influence of environmental factors on oocyte quality. In the present study, we examined the role of EGCG in delaying oocyte aging after ovulation in vitro. Ovulated oocytes were treated with EGCG at different concentrations, and the fragmentation rate, induced by aging, was significantly reduced by EGCG at 50 μM in vitro. To further elucidate the influence of EGCG on the quality of post‐ovulatory aged oocytes, we examined the spindle morphology of aged oocytes. The results showed that EGCG significantly decreased the abnormal rate of spindle morphology in post‐ovulatory aged oocytes. The high level of reactive oxygen species (ROS) and mitochondrial dysfunction in post‐ovulatory aged oocytes were also improved by EGCG, which might be a reason for the reduced apoptosis. The reduced sperm binding capacity of aged oocytes was increased by the addition of EGCG. These suggest that EGCG can improve the quality of post‐ovulatory aged oocytes.

## Graphical abstract

In this study, we find that EGCG delays post‐ovulatory oocyte aging and improves the quality of aged oocytes. EGCG may reduce ROS levels in aged oocytes by the NRF2‐KEAP1 pathway. The embryo developmental potential of aged oocytes is also increased by EGCG.

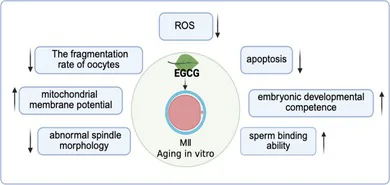

## 1 Introduction

Oocyte quality can be affected by chronic diseases, environmental pollutants, age, and gene mutations. Low oocyte quality can lead to subfertility/infertility in clinic. The ovulated oocyte begins to age if it cannot be fertilized timely. With aging, the oocyte developmental potential begins to decrease, such as low fertilization rate, mitochondrial dysfunction, abnormal spindle morphology and chromosome organization, increased accumulation of reactive oxygen species (ROS) level, and reduced embryo development (Nisio et al. 2022). The aged oocyte also have adverse impact on offspring health, such as mental abnormalities, increased hyperactivity, reduced fertility, and shortened lifespan (Tarín et al. 2002; Wilcox et al. 1998). Therefore, it is a crucial issue to delay post‐ovulatory oocyte aging in clinic.

Tea, a traditional beverage in China, has been drunk for thousands of years and become the most popular drink besides water worldwide. The health benefit of tea has been recorded since Tang and Song dynasties (Yang et al. 2014). In recent years, the role of tea in improving chronic diseases has been demonstrated by lots of studies, such as anti‐inflammation, anti‐obesity, anti‐bacterium, anti‐oxidation, and anti‐cancer (Dou 2019). The benefit of tea to health is mainly mediated by the bioactive components. Tea polyphenols, accounting for about 30%–40% of the dry weight of tea leaves (Zhang et al. 2024), are the most significant bioactive ingredients in tea (Yang et al. 2025). Numerous studies have demonstrated that tea polyphenols are beneficial for various diseases, such as cancer, chronic inflammation, diabetes, cardiovascular diseases, and liver diseases (Zhou et al. 2021). EGCG, the most abundant bioactive component of tea polyphenols (Liu et al. 2020), has function in antioxidant, scavenging free radical, and modulation of enzyme activities (Zhang et al. 2024). Zhao et al. have reviewed the benefit of tea to oocyte quality (Zhao et al. 2021). EGCG and tea ployphenols significantly improve the deleterious influence of maternal diabetes mellitus on oocyte quality in mice (Chao et al. 2023; Lu et al. 2022). EGCG also increases the fertility rates and embryo development of pig, and enhances the quality of oocytes from advanced females (HongHui et al. 2023). Nevertheless, the role of EGCG in delaying post‐ovulatory oocyte aging is not well known. Here, the role of EGCG in improving the quality of post‐ovulatory aged oocytes was examined, and we found that EGCG significantly increased the quality of post‐ovulatory aged oocytes in vitro.

## 2 Materials and Methods

### 2.1 Animals

SPF female and male ICR mice (6–7 weeks, 24–26 g) were purchased from Jinan Pengyue Laboratory Animal Breeding Co. Ltd. (Jinan; License No.: [2019]003146), and housed in a controlled environment with a light–dark cycle for 12 h at 23°C ± 2°C. Mice were fed ad libitum. This study was performed according to the People's Government of Shandong Province guidelines for the Care and Use of Laboratory Animals (no. [2018]311) and approved by the Ethic Committee of Qingdao Agricultural University (No. QAU20230510011).

### 2.2 Oocyte Collection and In Vitro Aging

Female mice were intraperitoneally injected gestational mare serum gonadotropin (PMSG) for 10 IU, and then injected human chorionic gonadotropin (hCG) for 10 IU at 46–48 h later. Mice were sacrificed at 13 h later after hCG, and cumulus‐oocyte complexes (COCs) were obtained and placed in M2 medium (CE003, MACGENE, Beijing, China). MII oocytes were randomly divided into two groups: aged and aged + EGCG. Fresh MII oocytes were used as control. Oocytes were aged for 24 or 48 h (Kazuo et al. 2004) in an incubator at 37°C with 5% CO2. EGCG (purity ≥ 98%) was purchased from Shanghai Macklin Biochemical Co. Ltd. (E808891, Shanghai, China) and dissolved into culture medium M16 for further experiments. Based on the previous studies (Chao et al. 2023; Lu et al. 2022), we further optimized the EGCG concentration at 25, 50, 75, and 100 μM, respectively. ML334 (MCE, HY‐110258, China) was used at 5 μM.

### 2.3 In Vitro Fertilization

Epididymal tails were isolated from male ICR mice and sperm were released into human tubal fluid (HTF). Spermatozoa were incubated at 37°C, 5% CO2 for 1.5 h. After that, oocytes were incubated with spermatozoa (1 × 10^−6^ per ml) in HTF at 37°C with 5% CO2 for 1 h. Oocytes were fixed with 4% paraformaldehyde (PFA) for 30 min and then transferred into mount with DAPI on slides. Results were examined by confocal microscope (Leica TCS SP5 II, Wentzler, Germany). To examine the embryo developmental potential, zygotes were incubated in KSOM (Sigma, MR‐106‐D) for 108 h.

### 2.4 Immunofluorescence

We fixed MII oocytes with 4% PFA for 30 min, and then permeabilized with 0.5% Triton X‐100 for 20 min. Thereafter, we blocked oocytes with PBS containing 1% BSA for 1 h. After that it was incubated with anti‐α‐tubulin FITC antibody (1:5000, T6199, Sigma‐Aldrich, MO, USA) or FITC anti‐mouse FR4 antibody (1:2000, 125,005, Bi‐legend, Beijing, China) overnight at 4°C. Secondary antibodies were stained for 2 h at room temperature. Following three washes, MII oocytes were transferred into mount with propylene iodide (PI) or DAPI. Fluorescence was examined with confocal microscope (Leica TCS SP5 II, Wenzler, Germany). DCF fluorescence was examined at 488 nm and DAPI fluorescence was examined at 405 nm.

### 2.5 Mitochondrial Membrane Potential Assay

The mitochondrial membrane potential was determined using the JC‐1 Mitochondrial Membrane Potential Assay Kit (C2006, Bessie Biotechnology Institute, Shanghai, China). Briefly, MII oocytes were transferred to JC‐1 working solution and incubated at 37°C with 5% CO2 for 20 min. After washing with JC‐1 buffer, fluorescence was examined by a fluorescence microscope (BX51, Olympus, Tokyo, Japan). DCF fluorescence was examined at 488 nm.

### 2.6 Ros

Intracellular ROS levels were measured with the ROS Assay Kit (S0033S Besse Biotechnology Institute, Shanghai, China). Briefly, MII oocytes were transferred to DCFH‐DA ROS assay solution and incubated at 37°C with 5% CO2 for 20 min. After washing, fluorescence images were captured using fluorescence microscope (BX51, Olympus, Tokyo, Japan).

### 2.7 Annexin‐ v Staining

Oocytes were stained with the Annexin V‐FITC/PI Apoptosis Detection Kit (A211‐02, Vazyme, Nanjing, China). Briefly, oocytes were washed twice with pre‐cooled PBS and incubated in binding buffer for 10 min (Li et al. 2023). After washing with binding buffer, fluorescence was examined with confocal microscope (Leica TCS SP5 II, Wenzler, Germany) at 488 nm.

### 2.8 Oocyte RNA Extraction and Relative Real‐Time PCR (qPCR)

Total RNA of oocytes was extracted with TIANGEN reagent (DP420, TIANGEN, Beijing, China) based on the manufacturer's instructions, and cDNA was synthesized using UnionScript First‐strand cDNA Synthesis Mix (Genesand, Beijing, China) according to the manufacturer's instructions. qPCR was performed at a 20 μL reaction system including 10 μL 2 × GS AntiQ qPCR SYBR Master Mix, 0.4 μL of each specific primers, 2 μL diluted cDNA template and 7.2 μL ddH2O. The 2^−ΔΔCt^ method was used to calculate the relative expression level of each gene, and GAPDH was used as an internal control.

### 2.9 Fluorescence Intensity Analysis

The average fluorescence intensity per unit area within the region of interest (ROI) was detected with Image J software (National Institutes of Health, MD).

### 2.10 Statistical Analysis

All percentage data were presented as mean ± SEM. Statistical analysis was performed using one‐way ANOVA and Tukey's test (*α* = 0.05). There were at least three biological repeats for each experiment. If *p* < 0.05, it was recognized as significant difference.

## 3 Results

### 3.1 EGCG Improves the Fragmentation Rate of Oocytes Undergoing Post‐Ovulatory Aging

**FIGURE 1:**
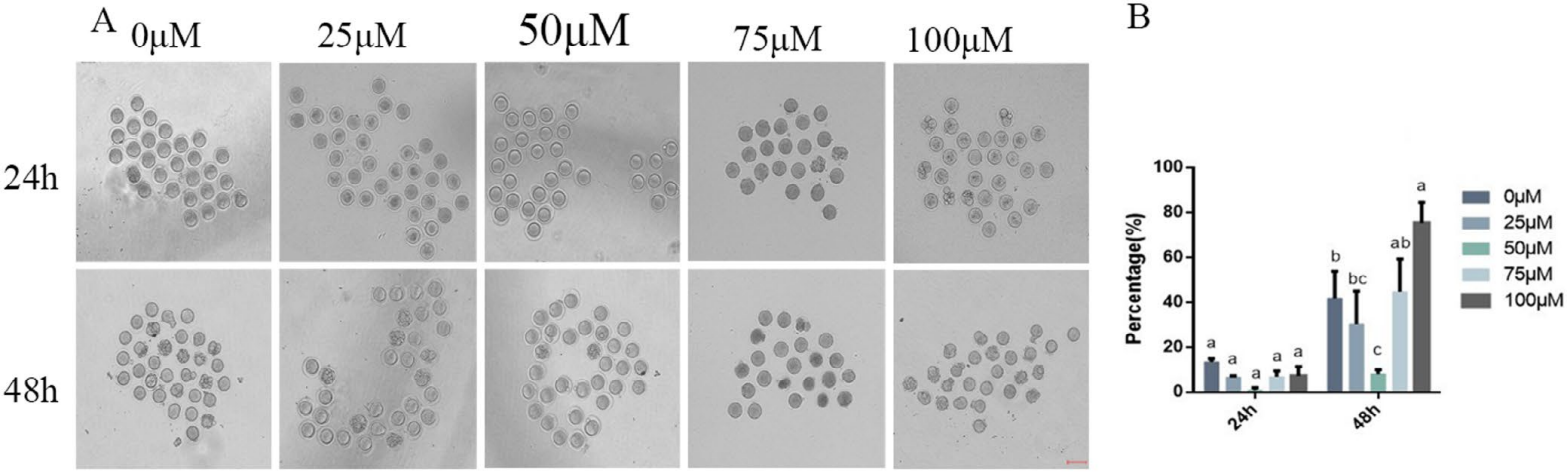
EGCG reduces the fragmentation rate of aged oocytes after ovulation. (A) Representative pictures of oocytes, respectively, aged 24 and 48 h with EGCG at 0 μM (*n* = 162), 25 μM (*n* = 156), 50 μM (*n* = 159), 75 μM (*n* = 120), and 100 μM (*n* = 157). (B) Fragmentation rate of oocytes was calculated at different aging times. Data were presented as mean ± SEM, with at least three independent experiments. The same latter means no significant difference between groups, and the different latter means significant difference between groups (*p* < 0.05). Bar, 100 μm.

To verify whether EGCG can delay post‐ovulatory oocyte aging, we added EGCG into the culture medium at concentrations of 0, 25, 50, 75, and 100 μM, respectively. We examined the fragmentation rate of oocytes aged 24 h in vitro, and found that the higher fragmentation rate of aged oocytes was significantly decreased by EGCG (Figure 1 and Table S1). To further confirm the role of EGCG in delaying post‐ovulatory oocyte aging, the aging time was extended to 48 h, and results showed that EGCG at 50 μM significantly decreased the fragmentation rate of post‐ovulatory aged oocytes (Figure 1A,B, and Table S1). These results suggest that EGCG can significantly delay post‐ovulatory oocyte aging in vitro at 50 μM. Therefore, EGCG at 50 μM and 24 h aging time were used for further experiments.

### 3.2 EGCG Restores Abnormal Spindle Morphology of Aged Oocytes

**FIGURE 2:**
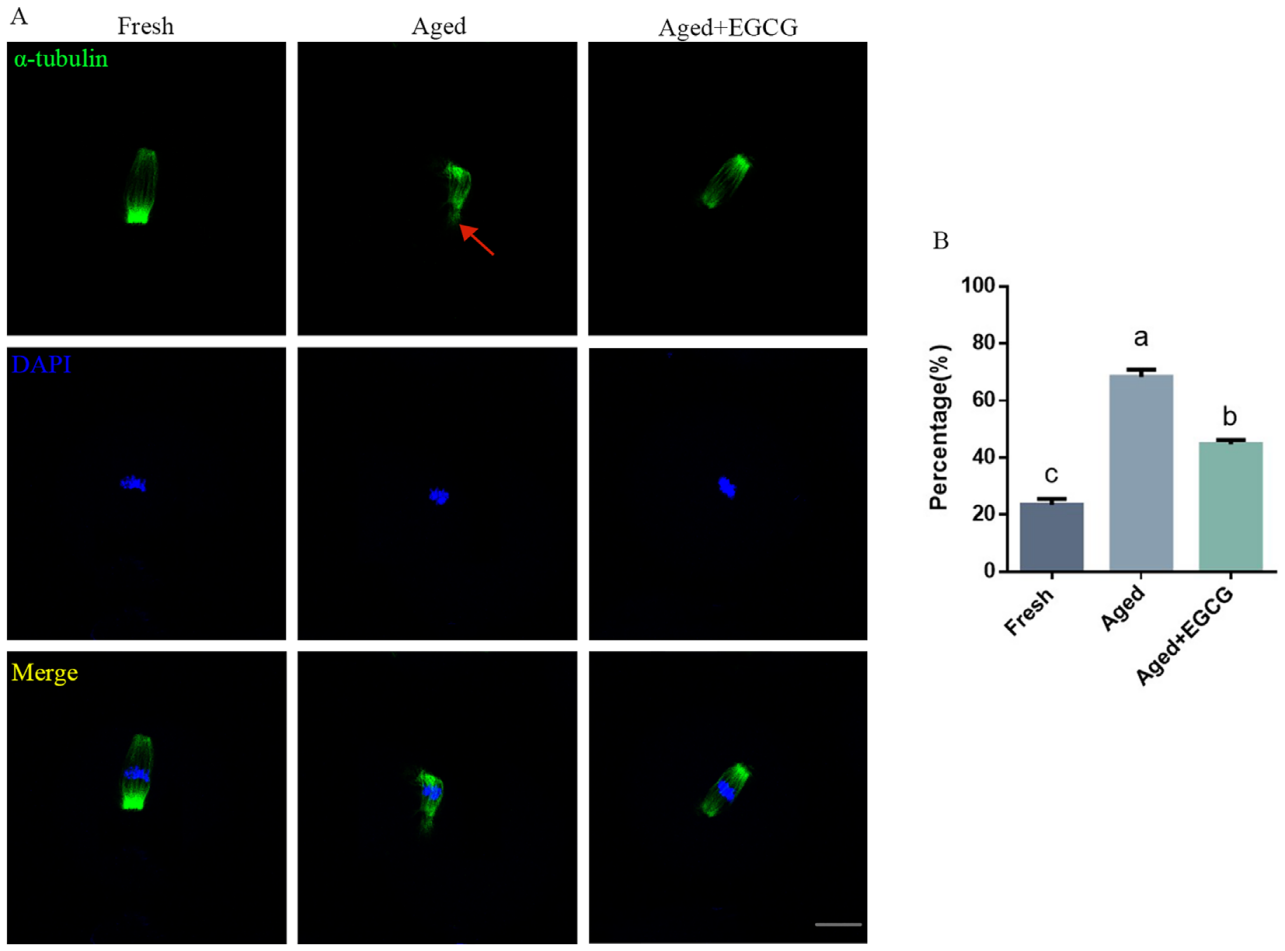
Effects of EGCG on spindle morphology of post‐ovulatory aged oocytes. (A) Representative pictures of spindle morphology in groups. Red arrow: Abnormal spindle; Scale bar = 15 μm. (B) Proportion of oocytes with abnormal spindle in the fresh (*n* = 104), aged (*n* = 95), and aged +EGCG (*n* = 102) groups. Data were presented as mean ± SEM, and repeated at least three times. The same letter means no significant difference between groups, and the different letter means significant difference between groups (*p* < 0.05). Bar, 500 μm.

Normal spindle morphology is crucial for chromosome segregation during meiosis. We found that post‐ovulatory aging significantly increased the abnormal rate of spindle morphology in oocytes compared with fresh oocytes (Figure 2). To investigate whether EGCG could improve the spindle morphology in post‐ovulatory aged oocytes, 50 μM EGCG was added. Results showed that the abnormal rate of spindle morphology in post‐ovulatory aged oocytes was significantly reduced by EGCG (Figure 2A,B, and Table S2). These suggest that EGCG can restore the adverse effects of post‐ovulatory aging on spindle morphology of oocytes.

### 3.3 EGCG Alleviates the Mitochondrial Membrane Potential of Aged Oocytes

**FIGURE 3:**
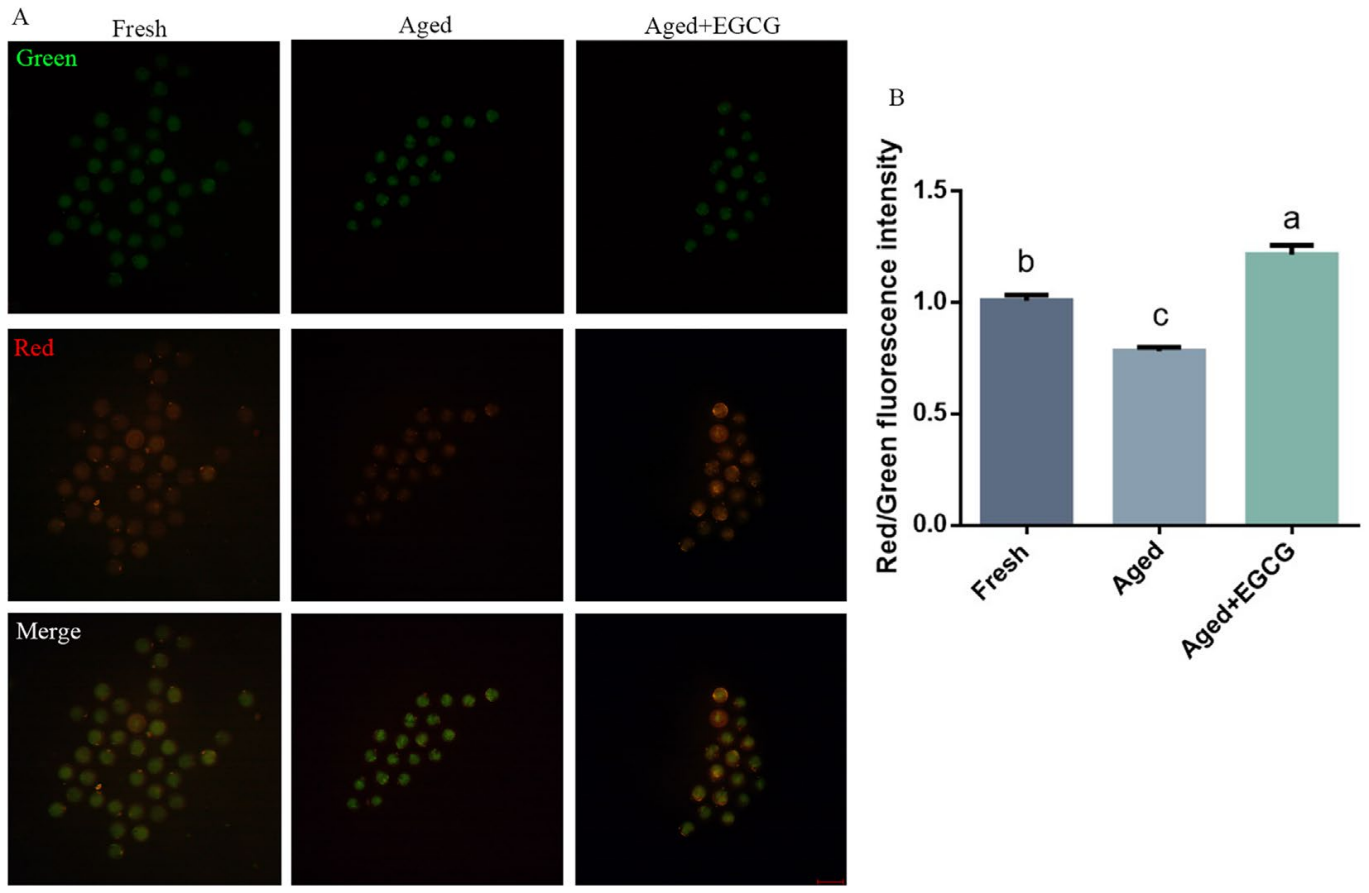
EGCG increases the mitochondrial function in post‐ovulatory senescent oocytes. (A) Representative pictures of the mitochondrial membrane potential examined by JC‐1 staining in groups. (B) Ratio of red/green fluorescence intensity in oocytes between groups (*n*, fresh vs. aged vs. aged + EGCG = 156 vs. 132 vs. 135). Data were presented as mean ± SEM, and repeated at least three times. The same latter means no significant difference between groups, and the different latter means significant difference between groups (*p* < 0.05). Bar, 100 μm.

Mitochondira, as the energy factories of cells, are abundant in oocyte, which provide ATP for various cellular activities and play a regulatory role in embryonic development (Thouas et al. 2006; Rémi et al. 2004). In this study, mitochondrial membrane potential was examined using the JC‐1 staining. Red fluorescence indicates high membrane potential, while green fluorescence means low membrane potential (Dai et al. 2015). The results indicated that the red‐to‐green signal ratio in post‐ovulatory aged oocytes was lower compared to the fresh group. After adding EGCG, the red‐to‐green signal ratio in post‐ovulatory aged oocytes was improved (Figure 3A,B, and Table S3). These indicate that EGCG can restore the mitochondrial dysfunction in aged oocytes.

### 3.4 EGCG Reduces the ROS Level in Aged Oocytes

**FIGURE 4:**
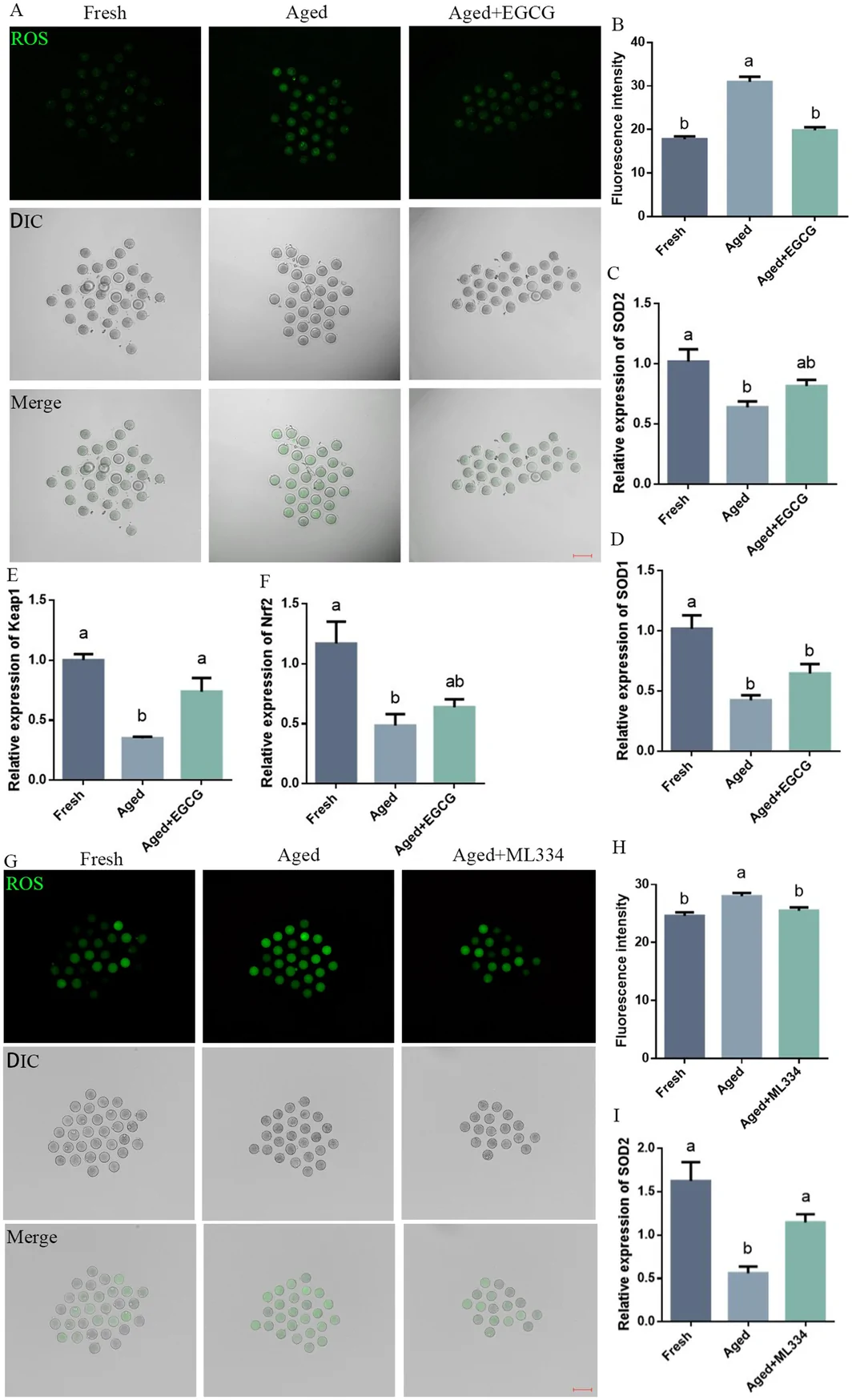
EGCG reduces the ROS level in post‐ovulatory senescent oocytes. (A) Representative pictures of ROS staining results in different groups. (B) Relative fluorescence intensity between groups (*n*, fresh vs. aged vs. aged + EGCG = 94 vs. 83 vs. 98). (C, D) The expression of *Sod2* and *Sod1* in oocytes. (E, F) The relative expression of *Keap1* and *Nrf2* in oocytes. (G, H) The representative pictures of ROS in groups and the relative fluorescence intensity in groups. (I) The relative expression of *Sod2* in oocytes treated with ML334. Data were presented as mean ± SEM with at least three independent repeats. The same latter means no significant difference between groups, and the different latter means significant difference between groups (*p* < 0.05). Bar, 100 μm.

Aging disrupts the balance between the ROS and antioxidant enzymes in oocytes, resulting in excess accumulation of ROS which is deleterious to oocyte potential (Ling et al. 2021). EGCG contains hydroxyl groups that play a key role in erasing ROS. Therefore, we examined whether EGCG could reduce ROS level in post‐ovulatory aged oocytes using DCFH fluorescent probe. Our results showed that the relative fluorescence intensity in post‐ovulatory aged oocytes was significantly higher than that in the fresh group. The addition of EGCG significantly reduced the ROS level in the post‐ovulatory aged oocytes, which was similar to the fresh group (Figure 4A,B, and Table S4). These suggest that EGCG can reduce the ROS level in post‐ovulatory aged oocytes. To elucidate how EGCG reduce ROS level, we examined the expression of superoxide dismutase (*Sod1* and *Sod2*) that can eliminate excessive ROS in cells (Lin et al. 2013). We found that the decreased expression of *Sod2* in post‐ovulatory aged oocytes was significantly increased by EGCG (Figure 4C). The relative expression of *Sod1* was increased by EGCG, but there was no statistical difference (Figure 4D). The expression of *Sod2* is regulated by the Kelch‐like ECH‐associated protein 1‐NF‐E2‐related factor 2 (NRF2‐KEAP) anti‐oxidative system (Yamamoto et al. 2018). We further examined the expression of *Keap1* and *Nrf2* in the aged oocytes. The results showed that the decreased expression of *Keap1* and *Nrf2* in post‐ovulatory aged oocytes was increased by EGCG (Figure 4E,F). To confirm the role of NRF2‐KEAP1 in aged oocytes, we activated the function of NRF2‐KEAP1 using ML334 during oocyte aging. The results showed that the ROS level in aged oocytes was significantly reduced by ML334 (Figure 4G,H), which may be mediated by the increase of *Sod2* (Figure 4I). These indicate that EGCG may reduce oxidative stress in post‐ovulatory aged oocytes via NRF2‐KEAP1 pathway.

### 3.5 EGCG Improves Apoptosis in Aged Oocytes

**FIGURE 5:**
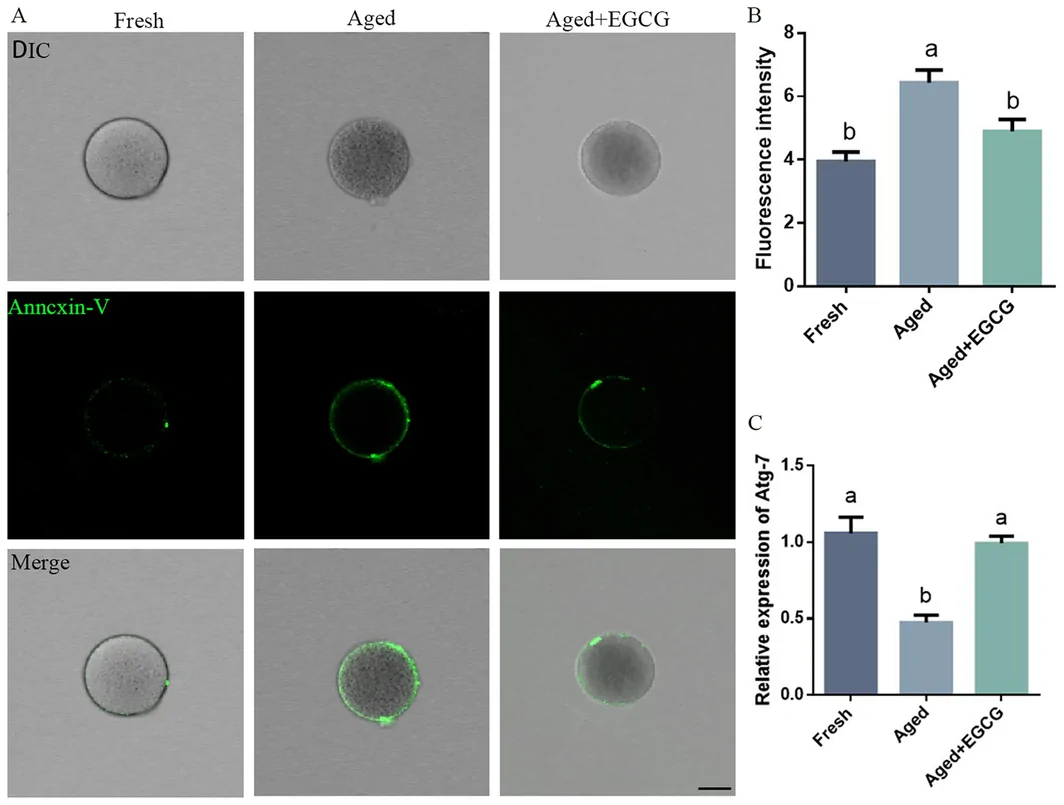
EGCG alleviates the apoptosis of post‐ovulatory senescent oocytes. (A) Representative pictures of apoptosis results in various groups (B) Relative fluorescence intensity statistics between various groups (*n*, fresh vs. aged vs. aged + EGCG = 101 vs. 83 vs. 81). (C) The relative expression of *Atg7* in oocytes. Data were presented as mean ± SEM, scale bar = 25 μm with at least three independent experiments performed. The same latter means no significant difference between groups, and the different latter means a significant difference between groups (*p* < 0.05). Bar, 50 μm.

Increased ROS levels and altered mitochondrial function in aged oocytes may induce apoptosis (Lord et al. 2015). Therefore, we examined the apoptosis of post‐ovulatory aged oocytes by Annexin‐V staining. We found that post‐ovulatory aged oocytes exhibited stronger fluorescent signals compared to that in fresh group. Notably, the addition of EGCG significantly reduced the fluorescent signals in post‐ovulatory aged oocytes (Figure 5A,B, and Table S5). These indicate that EGCG can ameliorate apoptosis in post‐ovulatory aged oocytes. Genes associated with autophagy such as autophagy related 7 (*Atg7*) regulate mitochondial function, oxidative stress and apoptosis (Baechler et al. 2019). To further investigate the mechanism, we examined the expression of *Atg7* by qPCR. Results showed that the expression level of *Atg7* was significantly down‐regulated in post‐ovulatory aged oocytes, which was significantly improved by EGCG treatment (Figure 5C). These findings suggest that EGCG might ameliorate apoptosis in post‐ovulatory aged oocytes by autophagy.

### 3.6 EGCG Alleviates the Impairs of Aging on Embryo Development

**FIGURE 6:**
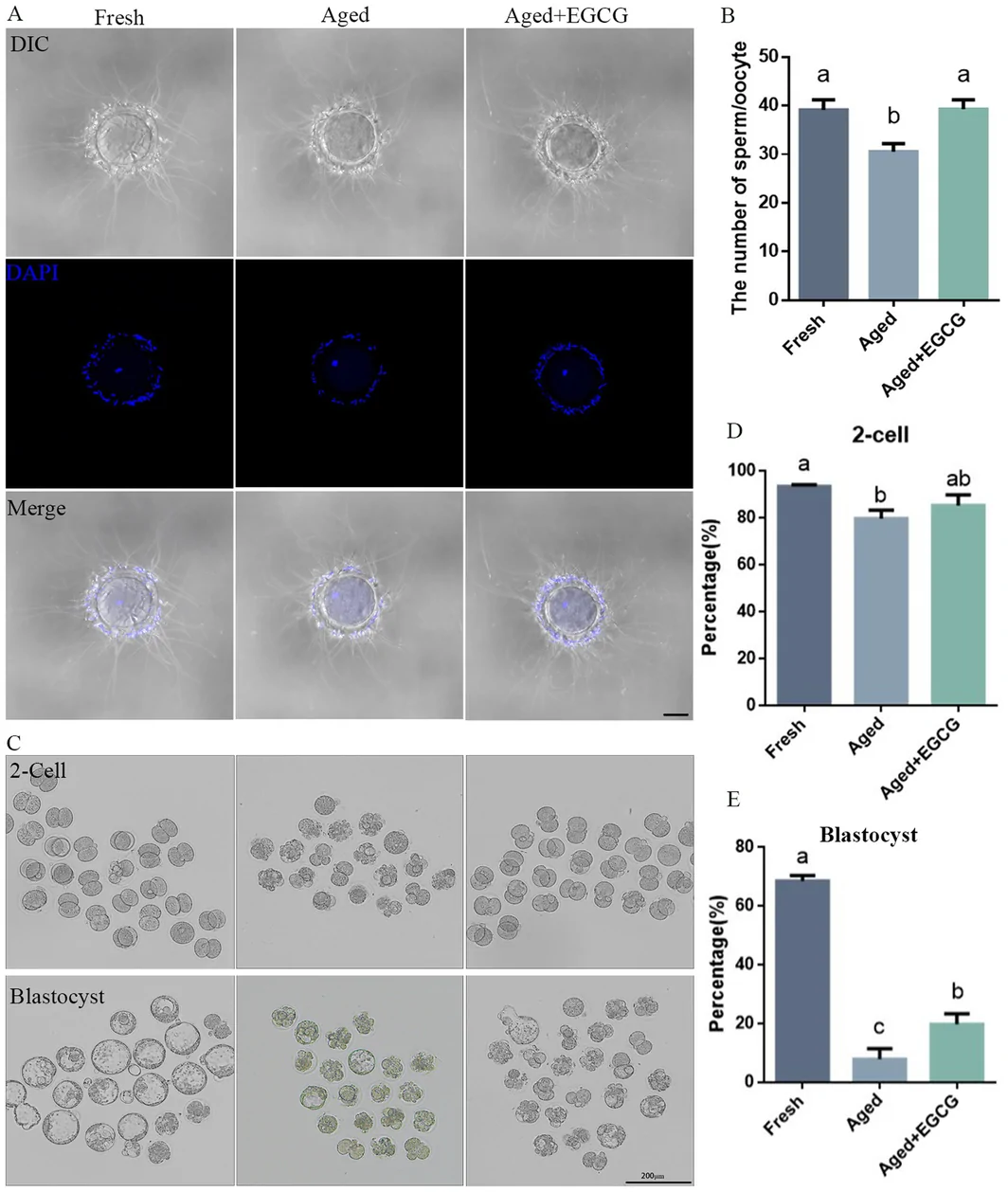
EGCG increases the embryo developmental potential. (A) Representative pictures of sperm binding capacity between various groups. (B) Sperm binding rate between various groups (*n*, fresh vs. aged vs. aged + EGCG = 58 vs. 43 vs. 60). (C) The representative pictures of embryos at 2‐cell and blastocyst stages in groups. (D, E) The embryo rate at 2‐cell and blastocyst stages in groups. Data were presented as mean ± SEM, scale bar = 25 μm with at least three independent experiments performed. The same latter means no significant difference between groups, and the different latter means significant difference between groups (*p* < 0.05).

Aging oocytes after ovulation undergo degenerative changes such as zona pellucida (ZP) hardening (Nogués et al. 2005). This affects the binding between oocytes and sperms. Therefore, we conducted tests to assess the sperm binding ability of post‐ovulatory aged oocytes. The results demonstrated that the sperm binding ability of aged oocytes after ovulation was significantly reduced compared to that of fresh oocytes (Figure 6). However, the addition of EGCG significantly improved the sperm binding ability of post‐ovulatory aged oocytes, which was similar to the fresh group (Figure 6A,B and Table S6). To further examine the embryo development, fertilized oocytes were cultured in vitro for 108 h. The results showed that the cleavage rate and the blastocyst rate were significantly lower in aged group compared with the fresh group, but it was improved by the addition of EGCG (Figure 6C–E). These indicates that EGCG can enhance the sperm binding ability and embryo developmental potential of post‐ovulatory aged oocytes.

## 4 Discussion

Mature oocytes have an optimal fertilization window after ovulation, and studies have shown that the optimal window for mice is 8–12 h (Chuanxin et al. 2022). In some conditions, the mature oocytes cannot be fertilized timely in vitro or in vivo, which begins to age. With the increase of aging time, the rapidly decreased oocyte quality leads to a bad pregnancy outcome (Kong et al. 2025). Therefore, delaying post‐ovulatory oocyte aging is crucial to improve the pregnancy outcomes. Here, we examined the role of EGCG in restoring the quality of post‐ovulatory aged oocytes, and found that EGCG significantly improved the aged oocyte quality.

Oxidative stress is a key initiating factor in the aging of mammalian oocytes after ovulation (Tessa and John 2013). The accumulation of ROS in aging oocytes may induce DNA damage and apoptosis (Hope et al. 2022). Natural components have been widely used to reduce the impacts of oxidative stress on health. Flavonoids protect neurons from oxidative damage by the c‐Jun N‐terminal kinase (Schroeter et al. 2001). Epicatechins reduces the impairs of hydrogen peroxide on human fibroblasts by inhibiting the oxidative stress (Spencer et al. 2001). Diosmetin delays porcine oocytes aging by improving the impaired mitochondrial function (Ren et al. 2025). Our previous studies indicate that tea polyphenols and EGCG can significantly reduce the ROS level of oocytes in diabetic mice (Chao et al. 2023; Lu et al. 2022). In the present study, we found that EGCG supplementation at 50 μM could significantly reduce the ROS level in aged oocytes. These indicate that EGCG can mitigate the oxidative stress in aged oocytes. The NRF2‐KEAP1 anti‐oxidative system (Yamamoto et al. 2018), serving as the master regulator of the antioxidant response element (ARE), controls the transcription of numerous cytoprotective genes. *Nrf2* deficiency typically leads to the down‐regulation of various antioxidant enzymes including SOD2 (Tonelli and Chio 2018), which is the major enzymes scavenging ROS in cells (Wang et al. 2026). During the oocyte maturation, equol, betulinic acid, and N‐acetylcysteine may alleviate the damage of excessive ROS on oocyte quality via NRF2‐KEAP1 pathway (Fan et al. 2022; Kim et al. 2024; Xiao et al. 2025). We found that the supplementation of EGCG effectively increased the expression of *Keap1* and *Sod2* in post‐ovulatory aged oocytes. When the NRF2‐KEAP1 was activated by ML334, the ROS level in aged oocytes was also reduced. These suggest that EGCG may reduce ROS level in aged oocytes via the NRF2‐KEAP1 anti‐oxidative system.

Mitochondria are essential for oocyte maturation and embryo development. Nevertheless, excessive ROS accumulation impairs mitochondrial function. It's reported that oocyte aging can induce structural abnormalities of mitochondria (Gümüş et al. 2010). We found that the reduced mitochondrial membrane potential in post‐ovulatory aged oocytes was ameliorated by the addition of EGCG. Previous studies have shown that mutations or damage to mitochondria can lead to spindle abnormalities and aneuploidy in oocytes (Eftekhari et al. 2021; Keefe et al. 2015). Morphological changes of the spindle in MII oocytes can further impact fertilization and embryonic development. Our study found that the addition of 50 μM EGCG during aging significantly reduced the spindle structural abnormalities.

Zona pellucida (ZP) is a semi‐permeable extracellular matrix surrounding oocyte. ZP consists of three or four glycosylated proteins, called ZP1‐4. During fertilization, ZP3 binds the plasma membrane of sperm and induces acrosome reaction (Reid et al. 2011). After that, the N‐terminal of ZP2 is cleaved which hardens the ZP to block polyspermy (Nishio et al. 2024). Post‐ovulatory aging induces ovastacin prematurely exocytosis in oocytes resulting in the cleavage of ZP2, which blocks the interaction between the sperm and oocyte (Xiaoxin et al. 2017). In the present study, we found that small number of sperm bound to the aged oocytes. However, the addition of 50 μM EGCG significantly increased the interaction between sperm and aged oocyte. These suggest that EGCG could increase the fertility of aged oocytes.

## 5 Conclusion

In a word, we find that EGCG improves the aged oocyte quality. However, there are still some limitations for this study. Although in vitro experiments are widely used to elucidate the life process, it cannot completely mimic the internal environment. In vivo experiments are needed before it can be used in clinic. The molecular mechanisms how EGCG delays oocyte aging are not well elucidated. The effects of EGCG on the pregnancy outcome of aged oocytes is still unclear. Therefore, more studies are necessary in the future.

## Author Contributions

**Jing Lin:** methodology, data curation, investigation, validation, formal analysis, writing – original draft. **Hua‐Chao Gao:** methodology, data curation, investigation. **Li‐Jun Li:** methodology, investigation. **Miao Hu:** methodology, investigation. **Li‐Fan Guo:** methodology, investigation. **Hong‐Yan Guo:** methodology, investigation. **Xiao‐Wen Jin:** methodology, investigation. **Peng Xu:** methodology, funding acquisition, writing – review and editing, data curation, investigation. **Zhao‐Jia Ge:** conceptualization, methodology, supervision, project administration, resources, funding acquisition, writing – original draft, writing – review and editing. **Lei Zhao:** conceptualization, formal analysis, funding acquisition, writing – review and editing, project administration, supervision.

## Funding

This research was funded by National Natural Science Foundation of China (32272775 and 32470566), the Central Government‐Guided Local Funds (YDZX2025039) and Science and Technology Vureau of Lvliang City (2023RC07) and Open Research Project of Biomedicine and Health Laboratory in Shanxi Province (BHLSXKF2026006).

## Ethics Statement

This study is supported by the Ethic Committee of Qingdao Agricultural University.

## Conflicts of Interest

The authors declare no conflicts of interest.

## Supporting information


**Table S1:** The extended data of Figure 1B.
**Table S2:** The extended data of Figure 2B.
**Table S3:** The extened data of Figure 3B.
**Table S4:** The extended data of Figure 4B.
**Table S5:** The extended data of Figure 5B.
**Table S6:** The extended data of Figure 6B.

## Data Availability

The data that support the findings of this study are available from the corresponding author upon reasonable request.
